# Asthma diagnosis and treatment – 1003. Severe asthma: a comparison of clinical severity and lung function

**DOI:** 10.1186/1939-4551-6-S1-P3

**Published:** 2013-04-23

**Authors:** Raja Dhar, AG Ghoshal

**Affiliations:** 1 Respiratory Medicine, National Allergy Asthma Bronchitis Institute, Kolkata, India

## Background

Severe therapy resistant asthma is a relatively rare entity. This has been variously defined based on clinical and spirometric criteria. It has always been known that the correlation between clinical and physiological parameters is not very consistent and hence the difficulty in defining this entity. We have looked at our cohort of consecutive patients with severe and moderate asthma in trying to establish a correlation between clinical and spirometric parameters.

## Methods

We looked at 850 consecutive patients with diagnosis of asthma based on Spirometric criteria (GINA guidelines). These were patients were compliance to medications was ensured and technique was optimised by trained healthcare personnel. We then selected the ones who had “Severe” & “Moderate” asthma basedon Spirometry. We now looked at these patients and correlated their Spirometry with thesymptomatology (use of rescue medication, night time awakening, exercise induced symptoms) and exacerbation rates (unscheduled GP visits, number of courses of OCS, emergency visits, hospitals and ICU admissions).

## Results

Only 79 (9.29%) patients out of 850 had severe asthma & 352 (41.41%) patients had moderate asthma based on Spirometric criteria. We found that Spirometric findings correlated with clinical parameters in 69.4% patients with Severe Asthma & 86% patients with Moderate Asthma.

## Conclusions

Only a small but significant percentage of patients in our population satisfy the Spirometric criteria of Severe Asthma (less than 10%). Even in this population there is lack of homogeneity between clinical parameters (symptoms & exacerbations) and physiological parameters (Spirometry). This correlation was much more consistent in patients with Moderate Asthma. Interestingly, we found more severe the asthma based on Spirometry, less was the correlation with symptomatology and exacerbations. This phenomenon and its causation needs to be studied in greater detail in longitudinal studies.

